# Double Cross-Linked Chitosan/Bacterial Cellulose Dressing with Self-Healable Ability

**DOI:** 10.3390/gels9100772

**Published:** 2023-09-22

**Authors:** Lili Deng, Kangkang Ou, Jiaxin Shen, Baoxiu Wang, Shiyan Chen, Huaping Wang, Song Gu

**Affiliations:** 1 State Key Laboratory for Modification of Chemical Fibers and Polymer Materials, College of Materials Science and Engineering, Donghua University, Shanghai 201620, China; 2 School of Chemistry and Chemical Engineering, Shanghai University of Engineering Science, Shanghai 201620, China; 3 Trauma Center, Shanghai General Hospital, Shanghai Jiao Tong University School of Medicine, Shanghai 201620, China

**Keywords:** methacrylated chitosan, dialdehyde bacterial cellulose, self-healing property, hydrogel, wound dressing

## Abstract

Self-healing hydrogel products have attracted a great deal of interest in wound healing due to their ability to repair their own structural damage. Herein, an all-natural self-healing hydrogel based on methacrylated chitosan (CSMA) and dialdehyde bacterial cellulose (DABC) is developed. MA is used to modify CS and obtain water-soluble biomaterial-based CSMA with photo crosslinking effects. BC is modified through a simple oxidation method to gain dialdehyde on the polymer chain. The success of the modification is confirmed via FTIR. Hydrogels are formed within 11 min through the establishment of a Schiff base between the amino of CSMA and the aldehyde of DABC. A dynamically reversible Schiff base bond endows hydrogel with good self-healing properties through macroscopic and microscopic observations. We observe the uniform and porous structure in the hydrogel using SEM images, and DABC nanofibers are found to be well distributed in the hydrogel. The compressive strength of the hydrogel is more than 20 kPa and the swelling rate sees over a 10-fold increase. In addition, the CSMA/DABC hydrogel has good cytocompatibility, with cell viability exceeding 90%. These results indicate that the all-natural self-healable CSMA/DABC hydrogel demonstrates strong application potential in wound healing and tissue repair.

## 1. Introduction

Skin is the largest organ in our bodies and protects us from external damage. A wound will form once the skin is injured. The wound repair process includes hemostasis, inflammation, proliferation, and remodeling [1]. Therefore, the wound repair process is complicated and difficult, especially for chronic wound or large-area skin tissue loss. Wound dressings can cover the wound and act as a physical barrier to prevent external infections [2]. It can also solve the problem of skin donor shortage. Therefore, wound dressing has become a mainstream treatment for promoting skin repair.

A variety of wound dressings have been developed, including both traditional and modern wound dressings. The desired wound dressings should meet the following requirements: good biocompatibility and good moisture retention, sufficient mechanical strength, and appropriate surface microstructure and biochemical properties [1]. Traditional dressings, such as gauzes and bandages, usually adhere to the wounds and cause secondary damage for patients [3]. More novel and functional wound dressings are being developed, like hydrogels, films and foams [4]. A moist environment has been demonstrated to facilitate wound healing [5]. Hydrogel dressings have three-dimensional (3D) porous structures, high water content, good mechanical properties, and biocompatibility. The moist environment provided by hydrogels conforms to the theory of moist healing. Hydrogel can be easily removed to avoid secondary damage. Therefore, hydrogels have become the most competitive alternative wound dressings.

General hydrogels are prone to breakage when exposed to external tension. Additionally, the fracture of hydrogels will lead to external the infection of wounds. So, it is important to maintain the structural integrity of hydrogels during wound healing. Therefore, self-healing hydrogel dressings that can repair their structural damage have attracted much attention in recent years. The healing mechanism of self-healing hydrogels includes chemical crosslinking (dynamic covalent reactions) and physical crosslinking (noncovalent reactions). Ionic bonding [6], hydrogen bonding [7], supramolecular interactions [8] and hydrophobic bonding [9] are usually utilized to prepare physical crosslinking hydrogels. A hydrogel composed of gelatin methacrylate and tannic acid was prepared and showed good self-healing property due to the presence of hydrogen bond [10]. However, compared to covalent bonding, noncovalent interactions usually lead to poor mechanical properties of hydrogels [11]. Chemical self-healing hydrogels based on dynamic covalent interactions are now attracting a lot of attention now. They can form original networks through acylhydrazone [12], disulfide, imines, Diels–Alder cycloadditon, and phenylboronate ester bonds. The desired self-healing hydrogels would autonomously and rapidly respond to damage and recover their original structure and mechanical strength [11]. Dynamic Schiff base (imine) linkages could achieve self-healing without external stimulus at a neutral pH [11]. Therefore, dynamic Schiff base (imine) linkages are usually applied to prepare self-healing hydrogel wound dressings [1,13,14]. In addition, natural material-based hydrogels are more advantageous in the biological field due to their excellent biocompatibility.

Chitosan (CS)- or hydrazide-modified polymers usually provide amino groups in dynamic Schiff base (imine) linkages. Synthetic polymers modified by benzaldehyde and oxidized polysaccharide often contribute aldehyde groups. CS is a natural polysaccharide and has antibacterial properties [15]. Additionally, it also has good degradability, biocompatibility, and immunity enhancement [16,17]. Therefore, it has been widely used in wound dressings due to its physiological functions. Huang et al. developed a hydrogel based on carboxymethyl chitosan and cellulose nanocrystal for deep partial-thickness burn wound healing. And the dynamic Schiff-base linkages endowed it with effective self-healing ability [18]. Zhang et al. prepared a self-healing hydrogel based on quaternized CS and benzaldehyde terminated PF127, and this showed a quick self-healing behavior [19]. The drawbacks of CS are that it is water-insoluble and has inadequate mechanical strength [20]. Moreover, methacrylated modified chitosan with water-soluble ability has been synthesized and can form hydrogel via photo-crosslinking [2,21]. Techniques such as forming double cross-linked networks or crosslinking with other polymers are usually utilized to strengthen the mechanical properties of CS.

Herein, we developed a double cross-linked hydrogel wound dressing with self-healing and improved mechanical properties using all-natural materials. The hydrogel was prepared based on two natural polymers including methacrylated chitosan (CSMA) and dialdehyde bacterial cellulose (DABC). CS was modified to CSMA by a normal method [21], giving it water solubility and photo-crosslinking abilities. Bacterial cellulose (BC) is synthesized by bacteria [22] and is also a natural polysaccharide. BC has a variety of desirable advantages, such as good mechanical properties, good biocompatibility, and high water-holding capacity [22]. Specially, BC has three-dimensional (3D) nanofibrous structure, which is similar to the structure to extracellular matrix (ECM) [23]. These characteristics endow it an ideal material in wound healing. In this paper. CSMA and DABC were firstly crosslinked by Schiff base reaction followed by photo-crosslinked to form double cross-linked hydrogel (CSMA/DABC). We characterized the chemical structure, physical morphology, mechanical property, self-healing ability, swelling ratio, and cytocompatibility of CSMA/DABC hydrogel. All the results showed that the self-healing hydrogel will be a good candidate for wound healing.

## 2. Results and Discussion

### 2.1. Chemical Structure Characterizations

**Figure 1:**
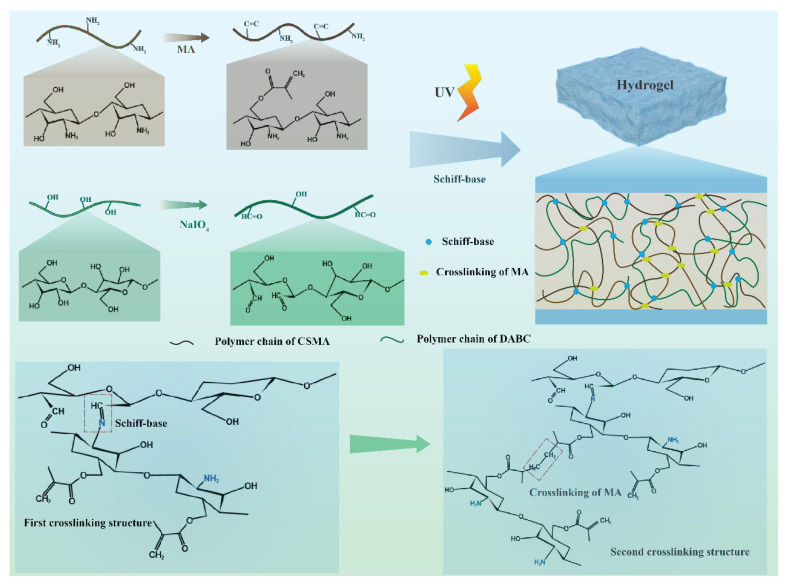
Schematic of CSMA/DABC hydrogel formation.

The preparation of self-healing hydrogels was based on methacrylate-modified chitosan and aldehyde-based bacterial cellulose (Figure 1). The amino groups of chitosan are partially replaced by photosensitive meth acrylamide groups. The hydroxyl groups on BC are oxidized and converted to aldehyde groups. The first crosslinking is formed by Schiff base reaction between amine groups of CSMA and aldehyde group of DABC. In the presence of photo initiators, the polymer chains of MA are able to crosslink with each other under light irradiation. In this way, the second network of hydrogel was constructed. The double crosslinking hydrogels present good mechanical properties.

**Figure 2:**
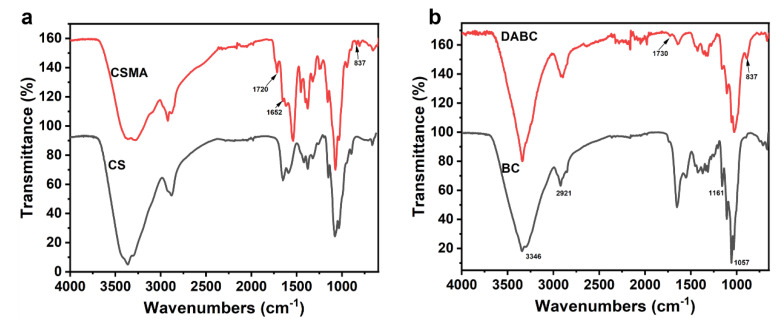
(**a**) FTIR spectra of CS and CSMA. (**b**) FTIR spectra of BC and DABC hydrogel.

The chemical structures of CS, CSMA, BC, DABC and CSMA/DABC were analyzed by FTIR. As shown in Figure 2a, the FTIR spectra of CS reveals the stretching bands for O–H and –NH_2_ (3331–3291 cm^−1^), stretching vibrations of C–H in –CH_2_OH and pyranose rings (2921–2877 cm^−1^), stretching vibrations of C=O group (1650 cm^−1^) and bending vibrations of N–H (1589 cm^−1^) [24]. For MACS, the characteristic peaks at 1720 cm^−1^, 1652 cm^−1^ and 837 cm^−1^ show the presence of C=O group and C=C group belonging to MA [2]. These results indicate the successful modification of CS. For BC, bands at 3346 cm^−1^, 2921 cm^−1^, 1161 cm^−1^ and 1057 cm^−1^ are corresponding to –OH, –CH, C–O–C and –CH_2_ stretching vibration, respectively (Figure 2b) [25]. After aldehyde, the characteristic absorption peaks of aldehyde group at 1730 cm^−1^ and 895 cm^−1^ are appeared, which are due to bending vibration of carbonyl and aldehyde –CH groups [26], respectively.

**Figure 3:**
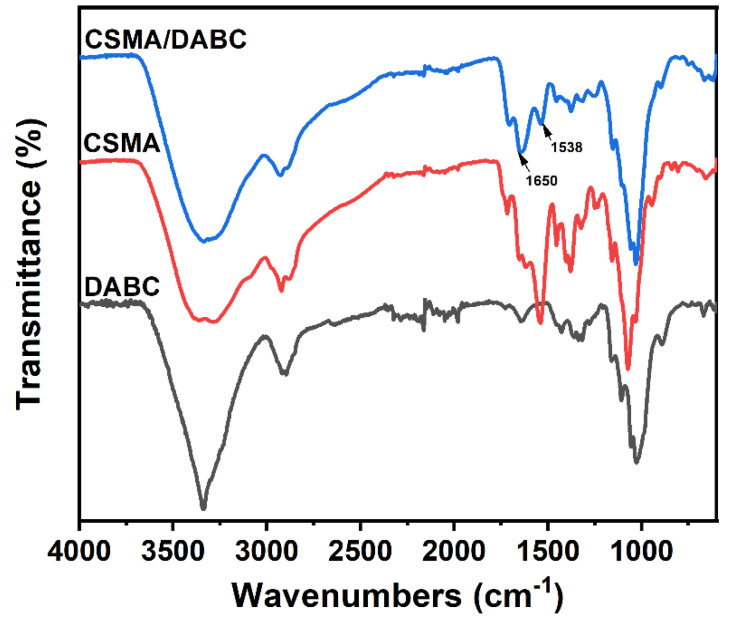
FTIR spectra of CSMA, DABC and CSMA/DABC.

After the cross-coupling reaction between CSMA and DABC, the amino and aldehyde groups combine to form a Schiff-base bond. The peak of –NH_2_ group at 1590 cm^−1^ on CSMA disappeared (Figure 3), as well as the characteristic peak of aldehyde group at 1730 cm^−1^, which implies the depletion of –NH_2_ and –CHO groups. In addition, the formation of the Schiff base bond is further confirmed by the stretching vibration peaks of the –C=N double bond at 1538 cm^−1^ and 1650 cm^−1^ [27]. These results suggest that the Schiff base reaction between –CHO in DABC and –NH_2_ in CSMA forms a dynamic crosslinked network.

### 2.2. The Gelation Time of CSMA/DABC Hydrogels

The gelation time of hydrogels is an important parameter in practical applications. When hydrogels are used to fill deep irregular wounds, slow gelation is required to ensure adequate filling of the wound. Table 1 records the different ratios of CSMA and DABC and gelation time of the hydrogel formation. We found that all proportions of CSMA and DABC are able to form hydrogels within 11 min of ultraviolet light. The gelation time of hydrogels is related to the ratio of CSMA to DABC. When the ratio of CSMA to DABC is equal to 1, CSMA/DABC–2 hydrogel has the shortest gel time of 7 min. When the ratio of CSMA to DABC is less than 1 (CSMA/DABC–1) or greater than 1 (CSMA/DABC–3), the gel time increases, which may be due to the high density of CSMA/DABC–2 that can reduce crosslinking time [28]. As such, the gel time can be effectively adjusted by adopting the proportion of CSMA/DABC to meet the practical requirements in different situations.

### 2.3. Morphology of CSMA/DABC Hydrogels

**Figure 4:**
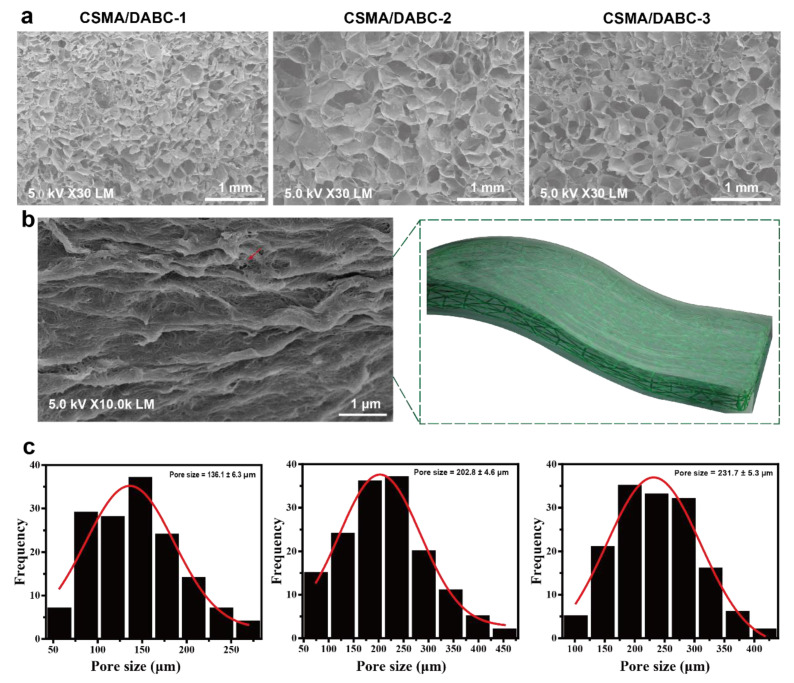
SEM images (**a**), magnification image (**b**) and average pore size (**c**) of CAMA/DABC hydrogels.

The morphology images were observed by FE-SEM. As shown in Figure 4a, hydrogels present a uniform and porous structure, and some DABC nanofibers are embedded in the hydrogel (Figure 4b, red arrow). With the increase of DABC content, the average pore size of the hydrogel gradually decreases from 231.7 μm to 136.1 μm (Figure 4c, red curve is the fitted curve). This may be ascribed to the presence of BC nanofibers. With the increase of DABC, the fiber bundles may be tangled to some extent, resulting in the gradual decrease in the pore size of the hydrogel [27]. On the other hand, the internal structure of the hydrogel becomes more compact as the DABC content increases. The controlled porous structure ensures that CSMA/DABC hydrogels have good water absorption, water retention and permeability, which will play a great potential in the field of medical dressings.

### 2.4. Swelling Performance

**Figure 5:**
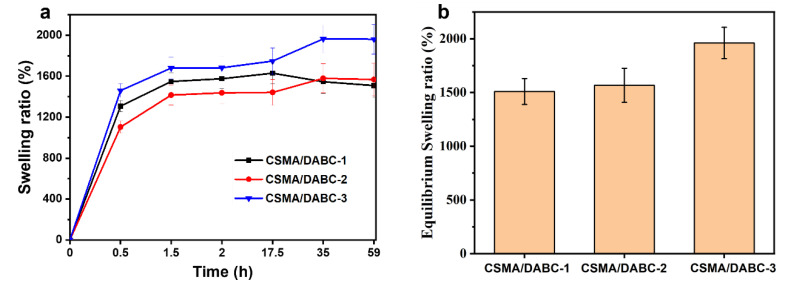
(**a**) Swelling ratio and (**b**) equilibrium swelling ratio of CSMA/DABC hydrogels.

The hydrogel reaches swelling equilibrium after absorbing water for about 60 h. As presented in Figure 5a,b, the swelling rate of CSMA/DABC–3 reaches the highest of about 2000% (a 20-fold increase), indicating it has the strongest water absorption capacity. Although the equilibrium swelling ratio of CSMA/DABC–1 hydrogel is the smallest, it also exceeds 1500%, higher than some of the report [29,30], indicating that CSMA/DABC has good swelling ability. The equilibrium swelling ratio is related to the internal network and pore size of the hydrogel. The CSMA/DABC–1 hydrogel owns the smallest pore size and the most compact internal network structure, which makes it difficult for water molecules to enter, resulting in a lower swelling ratio. In contrast, CSMA/DABC–3 hydrogel has the largest pore size and water molecules can enter more easily. This experiment proves that CSMA/DABC hydrogel has good swelling ability, can quickly absorb tissue exudate and maintain a moist environment in the wound.

### 2.5. Mechanical Properties

**Figure 6:**
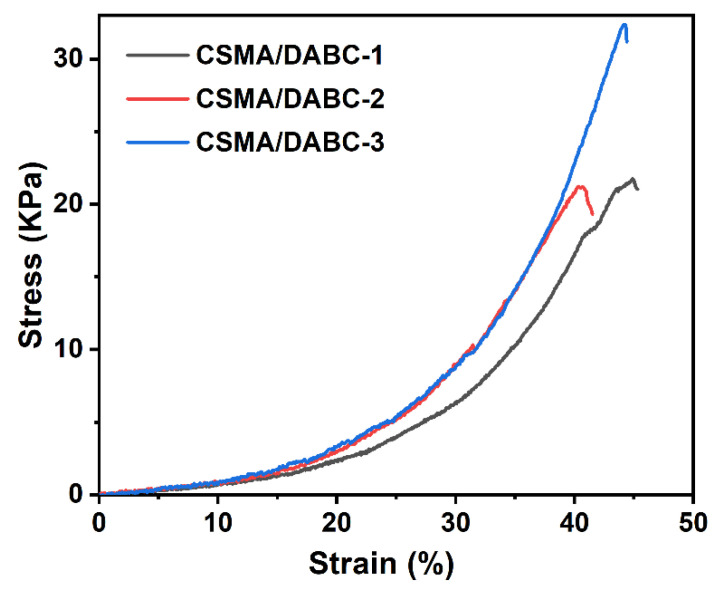
Compressive stress–strain curves of CSMA/DABC hydrogels.

The compressive stress-strain curve is presented in Figure 6 with a maximum compressive strain of 50%. Among them, the maximum compressive stress of CSMA/DABC–3 hydrogel reaches 33.7 kPa, which is mainly due to the double superposition of Schiff-base bond and methacryloyl chemical cross-linking in the hydrogel. The compressive performance of the double crosslinked structure is higher than those of the single Schiff-base bond [27]. When the CSMA content is low, Schiff base plays a dominant role, and the increase in CSMA content has little effect on the mechanical properties of the entire system. With the increase of CSMA content in the hydrogel, the crosslinked network of MA increases, which plays an important role in improving the compression strength.

### 2.6. Rheological Behaviors

**Figure 7:**
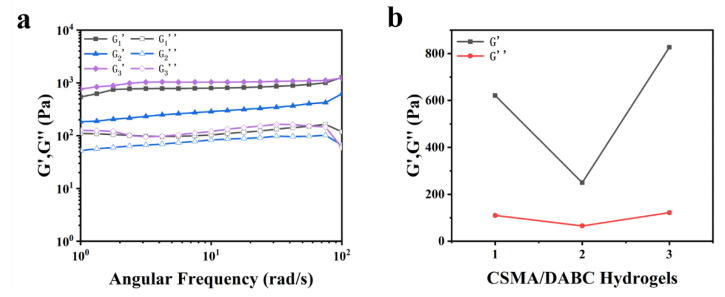
(**a**) G′, G″ curves of CSMA/DABC hydrogels with frequency sweep (ω = 1~100 rad/s) at a fixed strain (γ = 5.0%) at 25 °C. (**b**) G′, G″ values of CSMA/DABC hydrogels.

Figure 7a shows the storage modulus (G′) and loss modulus (G″) for a series of CSMA/DABC hydrogels with different ratios in the range of linear viscoelastic region. G′ is continuously higher than G″ in all hydrogels and G′ is smooth over the frequency range of ω = 1 to 100 rad/s, which demostrates that the material is in an elastic solid state with a stable network structure. We calculated their average values separately and plotted them in Figure 7b. It can be seen that G′ of CSMA/DABC–2 hydrogel is relatively low, which may be attributed to the fact that the internal crosslinking network of CSMA and the Schiff base network are interacting with each other, and the Schiff base bond plays a dominant role in this material system, which leads to a slight decrease in the storage modulus of the hydrogel compared with the other two hydrogels.

### 2.7. Self-Healing Properties

**Figure 8:**
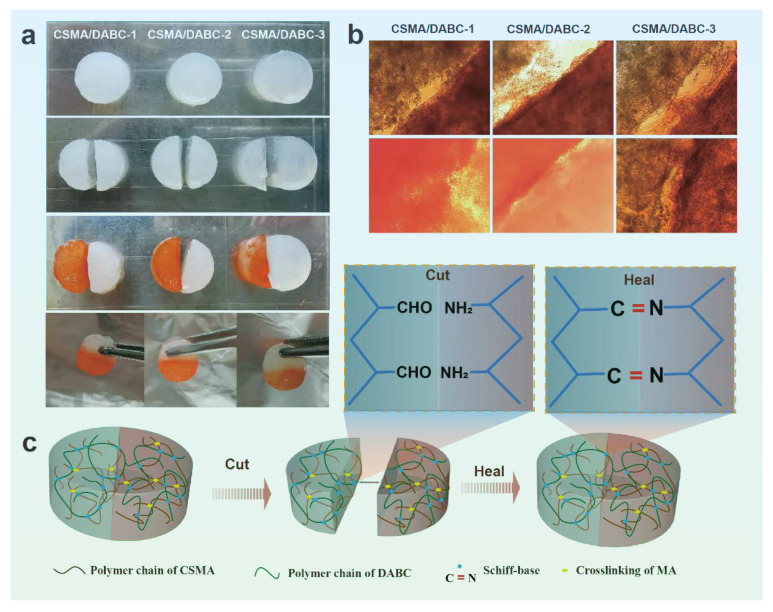
(**a**) From top to bottom: hydrogel photographs, cutting hydrogel photographs, dyed and healed hydrogel photographs. (**b**) Microscope photographs of the healed hydrogels. (**c**) Schematic illustration of the self-healing process.

Self-healing capabilities are critical for sports wounds as hydrogels are adaptable to wounds with a variety of injuries. The self-healing ability of hydrogels can greatly extend the life of the material [31]. The outstanding self-healing properties are presented in Figure 8. We prepared a series of CSMA/DACB hydrogels with different ratios and cut them into two halves from the middle, and the halves was stained with orange dye (Figure 8a). Subsequently, the two different parts are tightly fitted along the cut line for 2 h at room temperature to make full contact. Apparently, these hydrogels could be restored to the round shape as their initial state, and the healed hydrogels show good integrity after stretching them with tweezers. The whole process does not require any external intervention, which is much better than hydrogels that can only repair themselves under certain conditions. Through the images of the optical microscope (Figure 8b), we found that the hydrogel healed well and there are no obvious gaps between the healed hydrogels. These experiments demonstrate that CSMA/DABC hydrogels have good self-healing properties at room temperature. This is mainly attributed to the Schiff base bonds between the hydrogels, thich are reversible dynamic covalent bonds that can reconstruct the network of the hydrogels (Figure 8c) [31].

Self-healing hydrogels have been widely studied and researched in recent years. We summarize some articles on chitosan self-healing in recent years (Table 2) and compare their gelation time, compression properties, swelling properties, self-healing and adhesion properties. From the table, we can see that the prepared hydrogels show suitable gel time, excellent swelling ratio, good mechanical properties and self-healing performance. In short, the prepared CSMA/DABC hydrogels have good comprehensive performance.

### 2.8. Cytocompatibility Assessment

**Figure 9:**
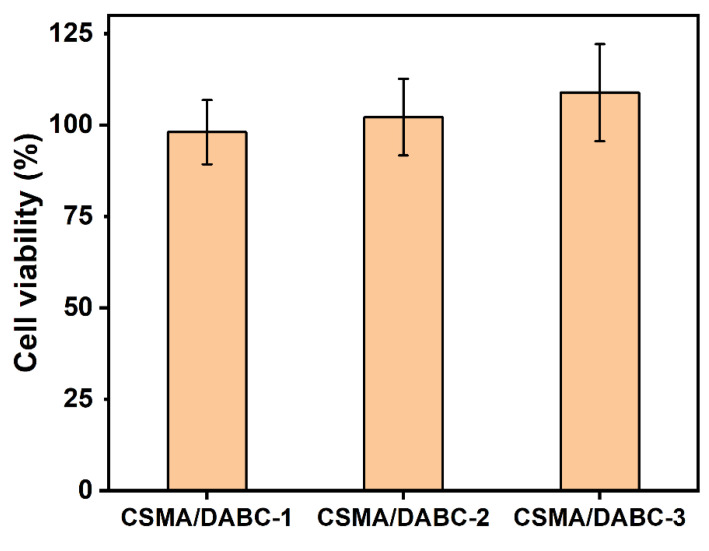
The viability of L929 cells cultured by hydrogel extracts.

Figure 9 presents the biocompatibility of the hydrogel. It is clear that the L929 cells activity also gradually increase with the increase of CSMA content. This may be due to the small amount of periodate residue in the DABC which has an effect on the cell activity. The cell viability of all hydrogels exceeds 90%, indicating that hydrogels have good biocompatibility. This is consistent with the biocompatibility of chitosan hydrogels [26]. Meanwhile, the cytotoxicity of hydrogels can be significantly reduced and the biocompatibility of the hydrogels can be improved by adjusting the composition of the materials.

## 3. Conclusions

In summary, we successfully developed a double cross-linked CSMA/DABC hydrogel with self-healing property for wound dressing. Specifically, we firstly synthesized methacrylated CS and aldehyded BC, and FTIR spectroscopy confirmed the success modification of CSMA and DABC, respectively. The CSMA/DABC hydrogel was then formulated via covalently crosslinking. The hydrogel consisted of two natural polymers, methacrylated CS and dialdehyde BC. We discussed the relationship between concentration ratio and gel time. By adjusting the proportion of CSMA and DABC, the gelation time, compression strength and swelling performance are optimized. The CSMA/DABC hydrogels exhibit self-healable properties due to the reversible Schiff-base. Meanwhile, the hydrogel shows good in vitro cytocompatibility in terms of L929 cell viability. Overall, the self-healing CSMA/DABC hydrogel is a promising wound dressing candidate for accelerating skin repair.

## 4. Experimental and Method

### 4.1. Materials

CS with a molecular weight of 100,000 Da (Deacetylation degree ≥ 85%) comes from Shanghai Jiachen Chemical Co., Ltd. (Shanghai, China). Sodium periodate and acetic acid were purchased from China National Pharmaceutical Group Chemical Reagent Co., Ltd. (Shanghai, China). Methylacrylic anhydride (MA) and phenyl (2,4,6-trimethylbenzoyl) lithium phosphinate (LAP) were purchased from Sigma Aldrich (St. Louis, MO, USA). Cell Count Kit 8 (CCK–8) is sourced from Dojindo Molecular Technologies (Rockville, MD, USA). L929 mouse fibroblasts were derived from the Shanghai Institute of Biochemistry and Cell Biology, Chinese Academy of Sciences, and were passaged by relevant personnel in our group.

### 4.2. Synthesis of CSMA

CSMA was synthesized according to previous paper [21]. Briefly, CS (3 g) was dissolved in 100 mL of 3 wt% acetic acid solution for 24 h. Then, methacrylic anhydride was dropped into this solution at a ratio of 3.5:1 (*w*/*w*, MA:CS) and reacted for 3 h. Then the reaction was stopped by equivalent volume of deionized water. The final solution was dialyzed for 5 days with 3–5 changes of deionized water per day. Finally, the dialyzed solution was freeze-dried for 24 h to obtain the CSMA.

### 4.3. Preparation of DABC

BC membranes were cultured in our lab as described previously [26]. The DABC was prepared according to our previous method [26]. Briefly, BC membranes were fibrillated into BC nanofibers by high-shear homogenization. Then BC nanofibers were dispersed in HCl solution (pH = 1) to form a homogeneous suspension. Sodium periodate (2.6 g) was added to the nanofibers (1.0 g) dispersion at 40 °C for 12 h. then the reaction was stopped by adding 2% ethylene glycol. The dispersions were further washed with deionized water to obtain DABC nanofibers.

### 4.4. Preparation of CSMA/DABC Hydrogels

CSMA was dissolved in deionized water to form a 2.5% wt/vol solution, and DABC was dispersed in phosphate buffered saline (PBS) to obtain a 2.5 wt% dispersion. HACC solution and DABC dispersions were mixed at ratios of 1:2, 1:1, 2:1. LAP (0.25 *w*/*v*%) was then added into the mixed solution. The hydrogel was obtained after thorough mixing and homogenization on an oscillator. CSMA/DABC hydrogels were firstly crosslinked through Schiff base reaction followed by UV light irradiation. The obtained hydrogels were named as CSMA/DABC–1, CSMA/DABC–2, CSMA/DABC–3, respectively.

### 4.5. Fourier Transform Infrared Spectroscopy (FTIR)

The resulting BC, CS, CSMA, DABC, and CSMA/DABC hydrogels were fully dried and then characterized by FTIR spectroscopy (Nicolet NEXUS–670, NICOLET. Madison, WI, USA) using ATR mode with a scanning range of 4000–600 cm^−1^.

### 4.6. Field Emission Scanning Electron Microscopy (FE-SEM)

The hydrogel samples are freeze–dried, then the samples are cut and attached to the conductive adhesive on the sample stage. The surface morphology of the samples was observed by FE-SEM (Hitachi S–4800, Hitachi High tech Company. Tokyo, Japan) at a voltage of 5 kV after the surface was plated with gold.

### 4.7. Gelation Time of Hydrogels

The inversion method was used to determine the gelation time of CSMA/DABC hydrogels. The CSMA solution was mixed with the DABC solution (1.5 g of gel precursor solution) in a 5 mL centrifuge tube and mix evenly on the oscillator. The tube was inverted repeatedly until it stopped flowing and became a complete gel, and the time was recorded with a stopwatch.

### 4.8. Swelling Ratio

Different ratios of CSMA/DABC hydrogel materials were tested for swelling properties. The CSMA/DABC hydrogel samples were thoroughly dried by freeze-drying and the mass (*M*_0_) was recorded. Then freeze-dried hydrogels were placed in ultrapure water for swelling at room temperature, and the mass of the hydrogel (*M_t_*) was recorded at specific time intervals. When the hydrogel absorbs water and the mass no longer changes, it means that hydrogels reach a constant weight (equilibrium swelling rate).

The swelling ratio (*E_sr_*) of a hydrogel can be calculated according to the following equation [37]:

(1) $$E_{sr}\;\left(\%\right)=\frac{M_{t}-M_{0}}{M_{t}}\times 100$$

### 4.9. Mechanical Performance Test

Mechanical compression tests of CSMA/DABC hydrogels with different ratios were carried out using Instron 5969 Double column Universal Testing System (Instron, Norwood, MA, USA). The samples were cylindrical with a diameter of 10 mm and a height of 10 mm. The samples were tested with compression rate of 5 mm/min and the maximum deformation of 50%.

### 4.10. Rheological Properties Test

CSMA/DABC hydrogels were characterized by a rotary rheometer (Anton Paar, Physica MCR301, Graz, Austria) with 25 mm diameter discs and a thickness of 3 mm. The test conditions as follows: at 25 °C, with a fixed strain of 5% and a scanning frequency of ω = 1–100 rad/s. The storage modulus (G′) and loss modulus (G″) of the hydrogels were determined.

### 4.11. Self-Healing Properties Measurements

CSMA/DABC hydrogels of 20 mm diameter and 1 mm thickness were prepared. The samples were cut into two semicircular hydrogels from the middle. One half-round hydrogel was stained with orange dye and the other half-round hydrogel was untreated. The two parts were then tightly affixed along the cut surface and left at room temperature. Then the two parts were tightly fitted together along the cut surface and left at room temperature. After 2 h, macroscopic and optical micrographs were taken to characterize the self-healing behavior of the CSMA/DABC hydrogels.

### 4.12. In Vitro Cell Toxicity

The mouse fibroblast (L929) was chosen to investigate the cytotoxicity through extract test method. First, CSMA/DABC hydrogels were sterilized in ethanol for 24 h. After sterilization, the samples were rinsed with sterile PBS to remove residual alcohol. The samples were then placed into 24-well plates and 2 mL of culture medium (containing 10% fetal bovine serum (FBS) and 1% penicillin-streptomycin (Invitrogen, Waltham, MA, USA)) was added to each well. Finally, the plates were incubated in incubator for 24 h to obtain the extracts. cells were seeded in a 96-well plate with four groups, three parallel samples per group. The cell density was 5 × 10^3^/well and incubated at 37 °C for 24 h. Then the obtained extracts (experimental group) were used for cell culture at 37 °C for 24 h. After that, 100 μL of CCK–8 reagent mix (CCK–8 reagent: serum-free medium = 1:9) was added into the 96-well plate and incubated for 90 min at 37 °C. Finally, a microplate reader (Infinite F50, Tecan, Männedorf, Switzerland) was used to detect the absorbance at 450 nm wavelength. The cytotoxicity was calculated as follows [24]:

(2) $$\mathrm{Cell}\mathrm{toxicity}\left(\%\right)=\frac{A_{s}-A_{o}}{A_{c}-A_{o}}\times 100$$

where *A_s_*, *A_o_*, and *A_c_* are the absorbance of the experimental group (cells cultured in the extract with CCK–8 mixed reagent), control group (cells cultured in medium, CCK–8 mixed reagent) and blank group (no cells, CCK–8 mixed reagent), respectively.

## Figures and Tables

**Table 1:** Gel time of CSMA/DABC hydrogels at room temperature (25 °C).

Hydrogel	CSMA (g)	DABC (g)	CSMA:DABC (g:g)	Gelation Time (min)
CSMA/DABC–1	0.5	1.0	1:2	10.5
CSMA/DABC–2	0.75	0.75	1:1	7
CSMA/DABC–3	1.0	0.5	2:1	9.25

**Table 2:** The properties compared to other chitosan self-healing references. (Notes: √ represents ‘have’, X represents ‘no have’).

Material	Gel Time	Swelling Ratio	Compression Property	Healing Mechanism	Adhesion Property	Date
Adenine-modified chitosan (AC) hydrogels [32]	Heating/cooling process	low	/	hydrogen bonding	X	2022
Quaternized chitosan-g-polyaniline (QCSP) and benzaldehyde group functionalized Poly(ethylene glycol)-co-poly(glycerol sebacate) (PEGS-FA) [29]	86–374 s	170–200%	/	Schiff base	√	2017
Tannic acid reinforced methacrylated chitosan/methacrylated silk fibroin [33]	20–90 s	ESR > 8	Compression resilience	Schiff base	X	2018
Fe, protocatechualdehy-de containing catechol and aldehyde and quaternized chitosan [30]	10–50 min	174–267%	2.2–15 N	Schiff base and catechol-Fe(III)	X	2021
CS, carboxymethyl-modified polymethyl methacrylate (PMAA) nanofibers and aldehyde sodium alginate [34]	145–366 s	/	/	Schiff base	X	2021
Chitosan and konjac glucomannan [28]	15–184 s	15–54%	/	Schiff base	√	2018
Tannic acid-reinforced metha-crylated chitosan/methacrylated silk fibroin hydrogels [2]	UV crosslinking	3.09–10.73%	25–108 kPa	X	√	2020
Peptide modified quaternized car-boxymethyl chitosan HTCC-P and oxidized dextran (OD) [35]	/	1960–2190%	/	Schiff base	X	2021
Water-soluble amidated pectin (AP) and oxidized chitosan [36]	50–170 s	255–558%	/	Schiff base	X	2021
Dialdehyde bacterial cellulose (DABC) nanofibers and chitoson [26]	53–78 s	/	/	Schiff base	X	2020
Quaternized chitosan and dialdehyde bacterial cellulose [27]	30–100 s	800–1600%	15–50 kPa	Schiff base	X	2022
This work	7–10.5 min	800–1900%	20–30 kPa	Schiff base	X	2023

## Data Availability

The data presented in this study are available on request from the corresponding author.
